# Characterization of Immunogenicity and Safety of COVID-19 mRNA-1273 in HIV-Positive Italian Patients with Hemophilia: A Prospective Single-Center Cohort Study

**DOI:** 10.3390/jcm12175475

**Published:** 2023-08-23

**Authors:** Chiara Suffritti, Roberta Gualtierotti, Sara Arcudi, Alessandro Ciavarella, Cristina Novembrino, Anna Lecchi, Silvia La Marca, Lidia Padovan, Erica Scalambrino, Marigrazia Clerici, Patrizia Bono, Ferruccio Ceriotti, Antonio Muscatello, Simona Maria Siboni, Flora Peyvandi

**Affiliations:** 1 Fondazione IRCCS Ca’ Granda Ospedale Maggiore Policlinico, Angelo Bianchi Bonomi Hemophilia and Thrombosis Center, 20122 Milan, Italy; 2 Department of Pathophysiology and Transplantation, Università degli Studi di Milano, 20122 Milan, Italy; 3 Department of Biomedical Sciences for Health, Università degli Studi di Milano, 20122 Milan, Italy; 4 Clinical Laboratory, Fondazione IRCCS Ca’ Granda Ospedale Maggiore Policlinico, 20122 Milan, Italy; 5 Infectious Diseases Unit, Fondazione IRCCS Ca’ Granda Ospedale Maggiore Policlinico, 20122 Milan, Italy

**Keywords:** hemophilia, vaccine, endothelial perturbation, COVID-19, coagulation, SARS-CoV-2

## Abstract

To characterize the immunogenicity of mRNA-1273 (Moderna, Cambridge, MA, USA) vaccine in HIV-positive hemophilic patients during the third COVID-19 wave in Italy and to investigate biomarkers of coagulation and endothelial perturbation before and after complete vaccination schedule, twenty-three consecutive adult HIV-positive patients with hemophilia were included. Blood was collected before and two weeks after vaccination. We measured anti-SARS-CoV-2 spike protein antibodies to assess immunogenicity; circulating biomarkers of coagulation (protein C and D-dimer), endothelial perturbation (von Willebrand factor (VWF)) and anti-Platelet Factor 4 (PF4) antibodies were analyzed. Flow-based analysis of thrombus formation was performed in nine patients using a flow-chamber device. Two weeks after completing the vaccination schedule, all patients had anti-spike antibodies values consistent with an effective immunization. Mean (±standard deviation) basal values of protein C and VWF (106 ± 21% and 171 ± 45%, respectively) were not significantly different from data obtained two weeks after the second dose (103 ± 20%, 162 ± 43%, respectively). D-dimer median values (interquartile range) were not significantly different at baseline (442 (603–142) ng/mL) and after the second dose (477 (654–262) ng/mL). Anti-PF4 antibodies were detected in three patients with no associated clinical manifestations. No significant differences were found in flow-based analysis of thrombus formation. Our data demonstrate that in HIV-positive patients with hemophilia, SARS-CoV-2 vaccination is effective and safe, with no effects on coagulation and endothelial perturbation.

## 1. Introduction

Severe acute respiratory syndrome coronavirus 2 (SARS-CoV-2) infection produces a complex picture of systemic and organ alterations collectively known as Coronavirus disease 2019 (COVID-19). The COVID-19 pandemic has proved difficult to manage and contain due to the biological nature of SARS-CoV-2 with high transmissibility and still partial knowledge of the pathogenetic mechanisms leading to severe and even lethal complications of the disease.

Currently, various COVID-19 vaccines have been developed, including mRNA vaccines based on the introduction of mRNA sequences for disease specific antigens [1]. On January 2021, the mRNA-1273 vaccine produced by Moderna received a conditional marketing authorization by the European Medicines Agency (EMA) Committee for Medicinal Products for Human Use (CHMP). In EMA’s Assessment Report [2], solicited local and systemic adverse reactions (AR) with an onset within seven days after each injection were reported. In the vaccine group of the pivotal phase 3 trial, 15,185 subjects were enrolled and 15,166 subjects were included in the placebo group. The local solicited ARs included pain, erythema, swelling, and lymphadenopathy. The general solicited ARs included fever, headache, fatigue, myalgia, arthralgia, chills, and nausea/vomiting. Regarding unsolicited adverse events (AEs), the incidences of unsolicited AEs, severe AEs, serious AEs (SAEs) and medically attended AEs (regardless of severity) were comparable between vaccine and placebo. In study participants who received the vaccine, three SAEs of cerebrovascular accident (one in placebo), two SAEs of embolic stroke (none in placebo), and one SAE of transient ischaemic attack (none in placebo) were reported. However, none of these events were considered as related to vaccination by the investigator as all subjects had a significant medical history or increased risk for these events. Two SAEs of deep vein thrombosis in the mRNA-1273 group were recorded (none in the placebo group), even if these events were not considered as treatment related.

The concerns caused by the report of SAEs after the administration of the vaccine could lead recipients to avoid the procedure itself.

Current guidelines suggest that the general recommendations regarding SARS-CoV-2 vaccination can be applied in patients with hemophilia as well [3,4,5,6,7]. Patients with human immunodeficiency virus (HIV) infection may be characterized by an increased risk of severe COVID-19 due to immunosuppression and higher rates of multimorbidity; therefore, these subjects may benefit from SARS-CoV-2 vaccination [8,9]. On the other hand, immune abnormalities, such as the exhaustion of B-cells, can occur in HIV-positive patients, particularly in those with uncontrolled persistent viral replication, leading to less durable long-term memory, and reduced capacity to adapt to new variants [10,11,12,13]. Overall, there is a lack of data on the efficacy and safety of COVID-19 vaccination in the group of patients with bleeding disorders and HIV, as the currently available data stem mainly from clinical trials including patients with HIV and no bleeding disorders [14,15]. Data regarding patients with HIV have been reviewed recently [16,17,18,19,20].

The aim of our study was to characterize the immunogenicity and safety of COVID-19 mRNA vaccine in HIV-positive hemophilic patients. In addition, we analyzed biomarkers of coagulation and endothelial perturbation before and after the administration of anti-SARS-CoV-2 vaccination.

## 2. Materials and Methods

### 2.1. Study Design

This was a single-center prospective cohort study conducted at Angelo Bianchi Bonomi Hemophilia and Thrombosis Center, Fondazione IRCCS Ca’ Granda Ospedale Maggiore Policlinico, Milan, Italy, from March to May 2021. The study was approved by the Istituto Nazionale per le Malattie Infettive Lazzaro Spallanzani IRCCS Ethics Committee (COVID-19: Perturbazione endoteliale, Emostasi, Risposta immuNItaria e del Complemento post VACCinazione”—Protocollo COPERNICO_VACC, PARERE N. 378.FAV del Registro delle Sperimentazioni 2020/2021) and was performed according to the 2013 revision of the Declaration of Helsinki and the code of Good Clinical Practice. The current study was reported following the Strengthening the Reporting of Observational Studies in Epidemiology (STROBE) Statement guidelines for reporting observational studies [21] and the Sex and Gender Equity in Research rationale for the SAGER guidelines and recommended use [22].

### 2.2. Patients

Consecutive adult HIV-positive patients with hemophilia followed at the Angelo Bianchi Bonomi Hemophilia and Thrombosis Center undergoing anti-SARS-CoV-2 vaccination were included. All participants provided written informed consent before enrollment. We collected blood before and two weeks after the administration of the complete vaccination schedule with mRNA-1273 (Moderna). The complete vaccination schedule consisted of two doses separated by an interval of six weeks.

Inclusion criteria: age ≥ 18 years, diagnosis of hemophilia and HIV infection. Exclusion criteria: age < 18 years, diagnosis of coagulation disorders other than hemophilia.

### 2.3. Laboratory Measurements

In order to assess immunogenicity, we measured antibodies to SARS-CoV-2 spike protein by Elecsys^®^ Anti-SARS-CoV-2 S (Roche, Basel, Switzerland), an electro-chemiluminescence immunoassay (ECLIA). The cut-off value for anti-Spike total Ig levels is 0.8 U/mL: higher values were considered positive.

Hematological parameters (whole blood parameters) and Immature Platelet Fraction (IPF), were analyzed in a Sysmex XN 1000 hematology analyzer (Sysmex, Kobe, Japan) equipped with a fluorescence channel for platelets counting. Values of CD3+ CD4+ (T Helper) were evaluated as part of the routine infectious disease follow-up visits within five months before vaccination.

Plasma levels of protein C and D-dimer were evaluated as biomarkers of coagulation and Von Willebrand factor (VWF) plasma levels as biomarkers of endothelial perturbation. Protein C was measured as chromogenic activity by means of the HemosIL Protein C kit (Werfen, Barcelona, Spain). D-Dimer was assessed by means of D-Dimer HS 500 HemoSil, an automated latex immunoassay (Werfen). VWF Antigen was measured using an automated latex enhanced immunoassay (HemosIL Von Willebrand Factor Antigen, Werfen). All the above tests were performed with the ACLTop 500 coagulation analyzer (Werfen). Anti-Platelet Factor 4 (PF4) antibodies were measured by means of ELISA (Werfen).

In a subgroup of patients free from any drug known to interfere with platelet function during the previous ten days, we analyzed flow-based thrombus formation with the Total Thrombus-Formation Analysis System (T-TAS) (Zacros, Fujimori Kogyo Co. Ltd., Tokyo, Japan). T-TAS is a flow-chamber device that analyses platelet-mediated thrombus formation in capillary channels through the following parameters: (1) the area under the flow-pressure curve (AUC), (2) occlusion start time (OST), the time needed to reach occlusion start-pressure, and (3) occlusion time (OT), the time needed to reach the occlusion pressure.

### 2.4. Statistical Methods

Based on data regarding antibody titer differences between basal and post vaccination values, we calculated that the study would require a sample size of 3 (number of pairs) to achieve a power of 80% and a level of significance of 5% (two sided).

We tested the Gaussian distribution with the Shapiro–Wilk test for each continuous variable. Continuous data were expressed as means ± standard deviation (SD) for normally distributed data. Median and interquartile ranges (IQR) were given for non-normally distributed data. To assess the statistical significance of the differences between groups, Student’s *t* test for paired values was used for normally distributed data, while Wilcoxon test was performed for non-normally distributed data. The data were analyzed using the SPSS statistical package, version 27 (IBM Corp. Released 2020. IBM SPSS Statistics for Windows, Version 27.0. Armonk, NY, USA: IBM Corp.), and a *p* value of <0.05 was considered statistically significant.

## 3. Results

We collected blood from 23 adult HIV-positive patients (22 males and one female, mean age 51.8, range 38–74) before and two weeks after the administration of the complete vaccination schedule. Eighteen patients were affected by hemophilia A, of which six were in prophylaxis with extended half-life (EHL) factor VIII (FVIII) (four with efmoroctocog alfa, two with damoctocog alfa pegol), seven on standard half-life (SHL) FVIII prophylaxis (four with moroctocog alfa, three with octocog alfa), three with episodic SHL FVIII (two with moroctocog alfa, one with octocog alfa) and two in prophylaxis with emicizumab; five patients were affected by hemophilia B, three of which were in prophylaxis with EHL factor IX (FIX) (two with eftrenonacog alfa, one with albutrepenonacog alfa) and two treated with episodic SHL nonacog alfa. Most patients had severe hemophilia (N = 20), while three patients had mild hemophilia. The majority of patients were diagnosed with HIV between 1983 and 1993 (19 patients), while four patients were diagnosed between 1994 and 2013. Patients started antiretroviral therapy on average 7.00 years after HIV diagnosis. The current antiretroviral regimen is reported in Table 1. The mean value of CD3+ CD4+ (T Helper) measured within five months before the first dose of vaccine was 722 ± 374 cells/µL (normal range 300–1400 cells/µL, minimum value in our cohort 307 cells/µL). No correlation was observed between the CD4+ count and antibody titer. HIV RNA was not detectable before vaccination in 18 patients, two patients had 20 copies/mL, one patient had 22 copies/mL while in two patients HIV RNA was not measured.

**Figure 1:**
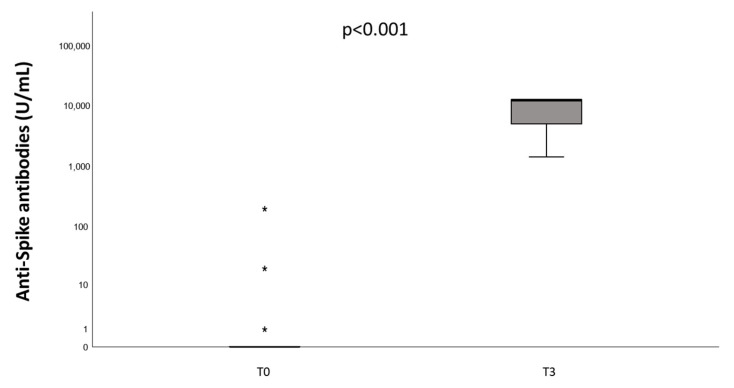
Anti-spike antibodies (U/mL) measured in patients with hemophilia undergoing COVID-19 vaccination at baseline (T0) and two weeks after the complete vaccination schedule (T3). Stars indicate values at a distance greater than 3 times the interquartile distance.

Before vaccination (T0), three patients out of 23 (13.0%) showed anti-Spike total Ig levels > 0.8 U/mL (cut-off value). Two weeks after completing the vaccination schedule (T3), all patients had values of anti-Spike total Ig compatible with effective immunization (median 12,116 U/mL, IQR 4496–12,500 U/mL, Figure 1). These values are higher in comparison with measurements obtained in 10 healthy donors 28 days after the second dose [23].

**Figure 2:**
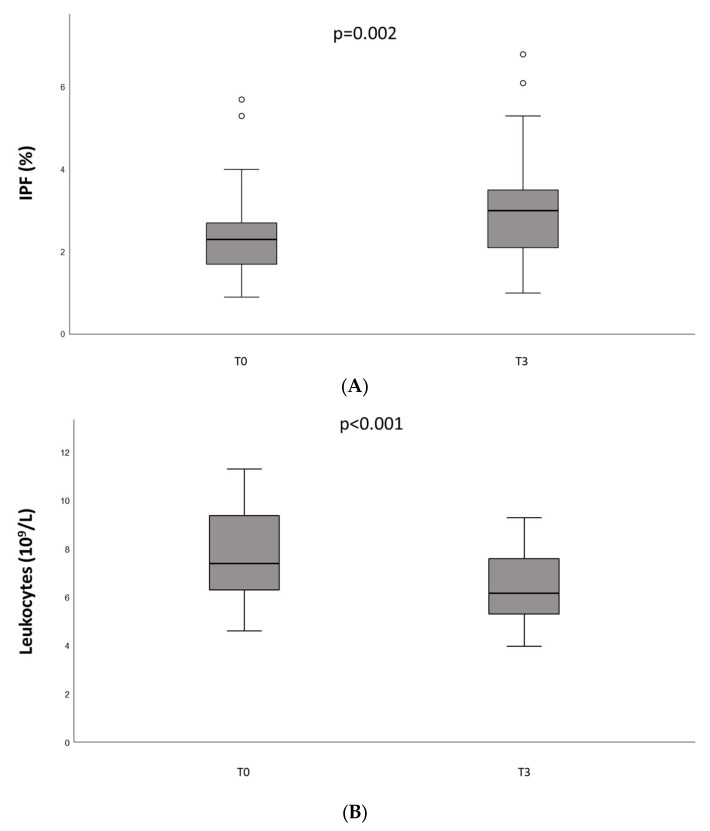

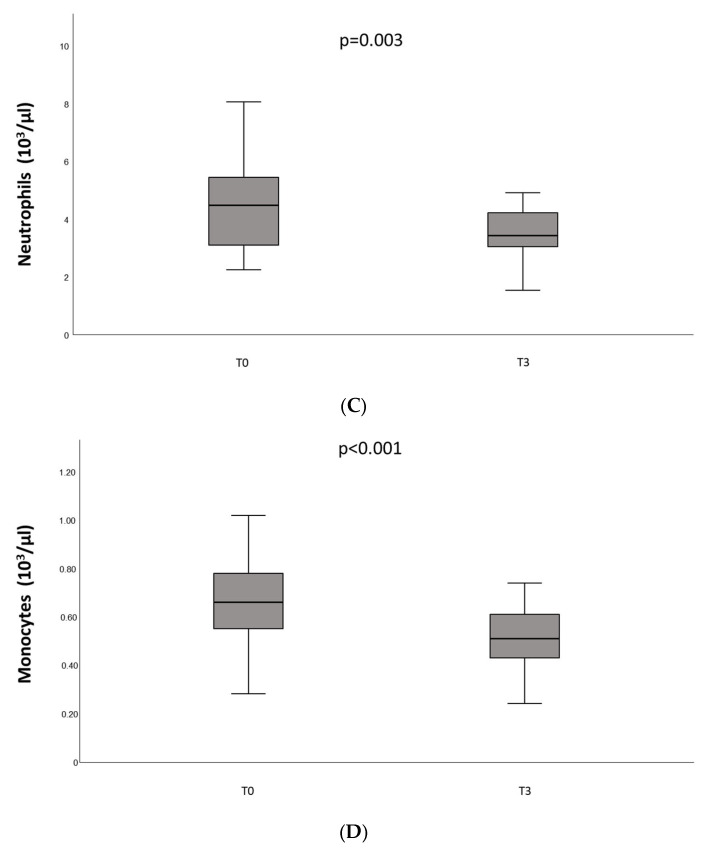
Immature platelet fraction (IPF, %) (**A**), leukocytes (**B**), neutrophils (**C**), and monocytes counts (**D**) measured in patients with hemophilia undergoing COVID-19 vaccination at baseline (T0) and two weeks after the complete vaccination schedule (T3). Circles indicate values at a distance greater than 1.5 times the interquartile distance.

Baseline platelet count was not significantly different from the value measured after vaccination (226 ± 74 10^9^/L vs. 233 ± 73 10^9^/L, *p* = 0.214). Significant differences were found for IPF (2.6 ± 1.3% vs. 3.2 ± 1.5% *p* = 0.002), leukocytes (7.81 ± 2.03 vs. 6.34 ± 1.52 10^9^/L *p* < 0.001), neutrophils (4.67 ± 1.76 10^3^/µL vs. 3.50 ± 0.81 10^3^/µL, *p* = 0.003) and monocytes (0.65 ± 0.21 10^3^/µL vs. 0.51 ± 0.14 10^3^/µL, *p* < 0.001) (Figure 2). No significant difference was observed in the lymphocyte count.

Concerning the T-TAS parameters, mean (± standard deviation) baseline values of AUC, OST, and OT, measured in a subgroup of patients free from drugs known to interfere with platelet function (N = 9), were not significantly different from values measured two weeks after the second dose of vaccine (310 ± 124 min×kPa, 2.1 ± 0.8 min, 6.6 ± 2.8 min, respectively vs. 306 ± 113 min×kPa, 2.4 ± 1.0 min, 7.1 ± 3.1 min).

**Figure 3:**
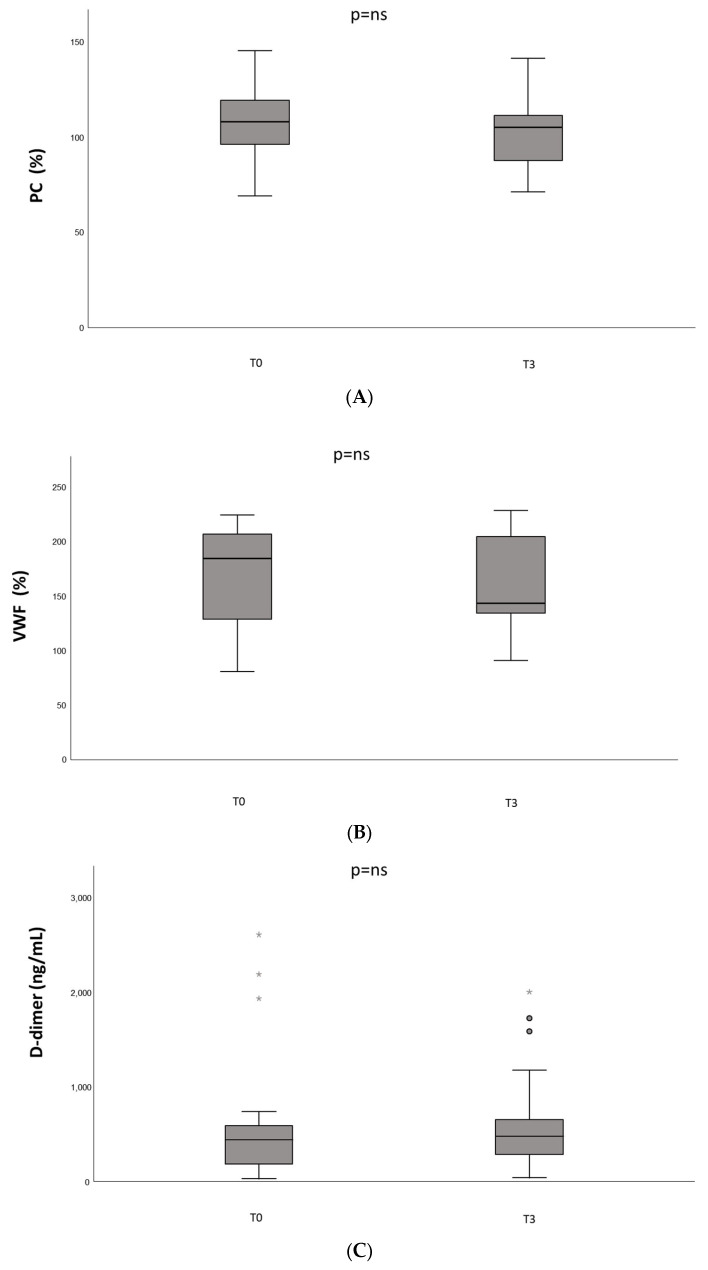
Plasma levels of biomarkers of coagulation and endothelial perturbation in the study population before (T0) and after 2 weeks from the complete vaccination schedule (T3): (**A**) protein C (PC) plasma levels (%). (**B**) Von Willebrand factor (VWF) plasma levels. (**C**) D-dimer plasma levels (ng/mL). Circles indicate values at a distance greater than 1.5 times the interquartile distance. Stars indicate values at a distance greater than 3 times the interquartile distance.

Mean (±standard deviation) basal values of protein C and VWF (106 ± 21%, 171 ± 45%, respectively) were not significantly different from values measured two weeks after the second dose of vaccine (103 ± 20%, 162 ± 43%). The D-dimer median basal value (IQR) was 442 (603–142) ng/mL and was not significantly different from the median value measured at T3 timepoint, 477 (654–262) ng/mL (Figure 3).

Since our population consisted of 22 males and one female, we checked whether the values of the variables in the female patient (who was affected by mild hemophilia B) differed from the mean values found in the male cohort (Table 2). We found that D-dimer and protein C plasma levels in the female patient before and after vaccination were lower than mean values in the rest of the population.

Anti-PF4 antibodies were detected in three patients (13.0%) at the end of the vaccination schedule with no associated clinical manifestations. None of the patients reported bleeding in the site of inoculation nor serious adverse events after the vaccination.

## 4. Discussion

Immune abnormalities can occur in HIV-positive patients, such as the exhaustion of B-cells [10,11,12]; consequently, it is important to collect data on COVID-19 vaccination immunogenicity. Recent studies demonstrated that people living with HIV with less than 200 CD4+ T-cells/µL have an impaired vaccine responsiveness [24,25]. In our population, all patients had CD4 cell counts included in the normal ranges. In HIV-positive patients with stable viral suppression and normal CD4+ T cell count, a normal humoral immune response is expected [24]. Our aim was to evaluate the stimulation of an adequate humoral response after SARS-CoV-2 vaccination and to assess the absence of bleeding or thrombotic complications in hemophilic patients with HIV.

We found that none of the patients reported bleeding in the site of inoculation or serious adverse events after the vaccination. We demonstrated that HIV-positive patients with hemophilia have a normal antibody response against SARS-CoV-2 spike protein. In addition, mRNA-1273 had no effect on coagulation and endothelial perturbation, as confirmed by values of protein C, VWF, D-dimer and T-TAS.

IPF is a measure of the youngest platelets in the peripheral circulation. It has been reported to be useful in the differential diagnosis thrombocytopenia, as IPF is high when peripheral consumption or destruction of platelets are increased such as in idiopathic thrombocytopenia purpura and thrombotic thrombocytopenia purpura and low in cases of bone marrow failure [26]. The significant difference found for IPF may reflect a stimulation on bone marrow that, however, also remains not clinically different considering the absolute number of platelets and the absence of a significant difference in platelet count before and after the complete vaccination schedule.

Sing et al. reported an increased risk of leukopenia among people receiving the BNT162b2 mRNA vaccine [27]. The mechanism causing leukopenia after BNT162b2 vaccination is not clear. Since decreased leukocyte counts after influenza vaccination were reported [28,29], it has been suggested that leukopenia could be due to a general immune response rather than to a vaccine-specific effect. In line with this hypothesis, our results showed that the leukocytes count decreased after vaccination, even if remaining within the normal range.

Gender medicine and inclusivity are increasingly considered in scientific research, based on the evidence that many normal physiological and pathological functions are influenced by sex-based differences [30] and that female patients are often underrepresented in clinical trials [22].

Hemophilia in female patients is very rare, but possible. Therefore, we decided to include in our study also a female patient with hemophilia. Although the data came from a single female patient, we found that D-dimer and protein C levels were lower compared with the rest of the population, which includes male subjects. This finding confirms the importance of collecting as much data as possible regarding female patients with hemophilia. Larger studies on this very rare population of subjects will help inclusivity and personalized medicine implementation.

This study has some limitations. The first limitation is the small number of the subjects included, although the sample size calculation has demonstrated that it was enough to reach statistical power. The study lacks a control group including HIV negative subjects and patients with hemophilia who are not living with HIV. In addition, the latest timepoint analyzed in our study was two weeks after the second vaccine dose. After a follow up of six months, no patient reported serious adverse events. In addition, data on the effectiveness of the vaccination are limited because of the short follow-up observation and the lack of data of SARS-CoV-2 infection confirmed by PCR molecular testing. Finally, we could not test the neutralization capacity of the antibodies. However, the titers were consistent with those observed in the healthy population.

## 5. Conclusions

In conclusion, SARS-CoV-2 vaccination is effective and safe in HIV-positive patients with hemophilia, with no severe thrombotic or hemorrhagic adverse effects and no signs of coagulation activation or endothelial perturbation.

## Figures and Tables

**Table 1:** Antiretroviral treatment regimen in the study population (N = 23).

Antiretroviral Treatment Regimen	Number of Patients (%)
darunavir + cobicistat + dolutegravir	5 (21.7)
dolutegravir + rilpivirine	3 (13.0)
bictegravir + emtricitabine + tenofovir	3 (13.0)
darunavir + cobicistat + emtricitabine + tenofovir	2 (8.7)
emtricitabine + tenofovir + raltegravir	2 (8.7)
dolutegravir + abacavir + lamivudine	1 (4.3)
darunavir + cobicistat	1 (4.3)
ritonavir + darunavir + emtricitabine + tenofovir	1 (4.3)
dolutegravir + ritonavir + darunavir	1 (4.3)
abacavir + lamivudine + nevirapine	1 (4.3)
lamivudine + zidovudine + dolutegravir	1 (4.3)
abacavir + lamivudine + efavirenz	1 (4.3)
emtricitabine + rilpivirine + tenofovir	1 (4.3)

**Table 2:** Values of the variables in the female patient compared with the mean values in the cohort. Values that differ from ranges are highlighted in bold.

	T0 (N = 22)	Female Patient T0	T3 (N = 22)	Female Patient T3
Protein C (%)	107 ± 21	**83**	103 ± 20	**79**
VWF (%)	171 ± 46	183	164 ± 44	139
D-dimer (ng/mL)	460 (625–203)	**28**	490 (693–299)	**40**
Platelets (10^9^/L)	223 ± 71	283	231 ± 74	274
IPF (%)	2.6 ± 1.3	3.1	3.1 ± 1.5	**5.0**
Leukocytes (10^9^/L)	7.64 ± 1.94	**10.24**	6.31 ± 1.55	7.08
Lymphocytes (10^3^/µL)	2.22 ± 0.89	**3.39**	2.10 ± 0.92	**3.40**
Neutrophils (10^3^/µL)	4.58 ± 1.70	5.85	3.52 ± 0.82	2.93
Monocytes (10^3^/µL)	0.65 ± 0.21	0.86	0.51 ± 0.14	0.64
Eosinophils (10^3^/µL)	0.14 ± 0.10	0.09	0.14 ± 0.08	0.07
Basophils (10^3^/µL)	0.04 ± 0.02	0.05	0.04 ± 0.02	0.04

VWF—Von Willebrand Factor, IPF—immature platelet fraction.

## Data Availability

The data presented in this study are available on request from the corresponding author. The data are not publicly available for privacy reasons.
